# Fragmenting the future with FLARE: a comprehensive fragmentomics pipeline based on long-read nanopore sequencing

**DOI:** 10.3389/fgene.2026.1794112

**Published:** 2026-03-13

**Authors:** Marica Ficorilli, Marta Lucchetta, Deborah Lenoci, Ilenia Rolli, Nicole Farina, Valentina Cristofaro, Lorenzo Giannini, Arianna Ottini, Alberto Deganello, Stefano Cavalieri, Lisa Licitra, Loris De Cecco

**Affiliations:** 1 Integrated Biology of Rare Tumors, Department of Experimental Oncology, Fondazione IRCCS Istituto Nazionale dei Tumori, Milan, Italy; 2 Head and Neck Medical Oncology Department, Fondazione IRCCS Istituto Nazionale dei Tumori di Milano, Milan, Italy; 3 Otolaryngology Head and Neck Surgery Department, Fondazione IRCCS Istituto Nazionale dei Tumori, Milan, Italy; 4 Department of Oncology and Hemato-Oncology, University of Milan, Milan, Italy

**Keywords:** cfDNA fragmentation, copy number profiling, flare, fragmentation and long-read analysis of regulatory epigenetics, fragmentomics pipeline, head and neck squamous cell carcinoma, HNSCC, long-read nanopore sequencing

## Abstract

**Purpose:**

Cell-free DNA (cfDNA) fragmentation patterns carry biological information beyond fragment length, revealing nuclease activity, chromatin organization, and tissue of origin. Fragmentomics has emerged as a powerful approach to improve circulating tumor DNA (ctDNA) detection, particularly at low tumor fractions. However, most current methods are designed for short-read sequencing, limiting their applicability to third-generation technologies. Here, we present FLARE (Fragmentation and Long-read Analysis of Regulatory Epigenetics), an integrated fragmentomics pipeline optimized for Oxford Nanopore long-read sequencing.

**Methods:**

FLARE preserves native cfDNA fragment ends and integrates copy number profiling, tumor fraction estimation, sequence-specific end-motif analysis, and methylation-based features to enable comprehensive characterization of cfDNA fragmentation. Plasma cfDNA from six patients with recurrent or metastatic head and neck squamous cell carcinoma (HNSCC) treated with nivolumab was analyzed at baseline (C1D1) and during therapy (C5D1).

**Results:**

Genome-wide copy number analysis revealed recurrent chromosomal alterations consistent with HNSCC biology, with ichorCNA-derived tumor fractions ranging from 0% to 12.8%. Tumor fraction estimates derived from methylation-based fragmentomic features showed concordant trends, providing an independent measure of tumor burden and correlating with clinical response. End-motif analysis based on 5′4-mer frequencies, combined with non-negative matrix factorization (NMF), identified predominant A-end and G-end patterns, consistent with apoptosis-associated nuclease activity.

**Conclusion:**

FLARE provides a robust and scalable framework for fragmentomic analysis using long-read sequencing, enabling simultaneous investigation of structural and sequence-level cfDNA features. This approach demonstrates the technical feasibility of integrated fragmentomic analyses on Nanopore cfDNA data and supports the future integration of native methylation and transcription factor binding site analyses.

## 1 Introduction

Cell-free DNA (cfDNA) consists of DNA fragments released into the bloodstream following cell apoptosis, necrosis, or secretion. In cancer patients, a subset of cfDNA is represented by circulating tumor DNA (ctDNA), which originates from tumor cells and carries essential genetic and epigenetic information (Tan et al., 2025). Recently, the field of fragmentomics (i.e., the study of cfDNA fragmentation patterns) has emerged as a powerful multi-omic approach to improve the sensitivity and specificity of ctDNA detection, even when tumor-derived fragments are present at very low fractions. For example, tumor-secreted DNA tends to be a shorter length compared to non-tumor-secreted DNA (Ding and Dennis Lo, 2022). Therefore, detecting the size of cfDNA fragments can improve the diagnosis of cancer patients compared to healthy subjects. However, the sensitivity of approaches based only on fragment size is still not sufficient for many clinical applications, especially in tumors with low ctDNA release rates. Growing evidence indicates that cfDNA fragmentation patterns are much more complex than length alone, including features such as nucleosome positioning, transcription factor binding footprints, and sequence-specific terminal motifs (Xu et al., 2024). These multidimensional signatures differ significantly between healthy individuals and cancer patients and provide complementary information to improve detection performance. Therefore, the development of a dedicated and comprehensive pipeline for fragmentomics is essential to fully unlock the potential of cfDNA as a biomarker in cancer research. Fragmentation features, such as end-motif patterns, nucleosome-associated signals, and methylation profiles, encode both tissue-specific and disease-related information (Tsui et al., 2025). However, their accurate extraction and interpretation require advanced computational tools, especially when using third-generation sequencing technologies such as Oxford Nanopore Technologies (ONT), which introduce unique analytical challenges but also unprecedented opportunities (Ficorilli et al., 2025). ONT enables fast, long-read sequencing of cfDNA while preserving native fragment structure and directly detecting epigenetic modifications without PCR or bisulfite conversion (Tsui et al., 2025). Compared to short-read sequencing, it captures a wider range of fragment lengths and provides more accurate methylation profiles. Combining fragmentation features with native methylation signals enhances the sensitivity and robustness of cancer detection and improves tissue-of-origin inference. A robust and optimized fragmentomics pipeline not only enables precise and scalable data analysis but also establishes a foundation for innovative approaches in non-invasive cancer detection, disease monitoring, and personalized therapeutic strategies. Liquid biopsy analysis has emerged as a valuable tool for monitoring immunotherapy efficacy in head and neck squamous cell carcinoma (HNSCC) (Chantre-Justino et al., 2022). Here, we present FLARE (Fragmentation and Long-read Analysis of Regulatory Epigenetics), an integrated fragmentomics pipeline optimized for Oxford Nanopore long-read sequencing. As a case study, we analyzed plasma samples collected within a clinical study involving patients with recurrent/metastatic (RM) head and neck squamous cell carcinoma (HNSCC) treated with immune checkpoint inhibitors. Following the introduction of PD-1 inhibitors, such as pembrolizumab and nivolumab, as standard treatments for RM HNSCC (Llop et al., 2025), several studies have investigated ctDNA kinetics as a correlate of treatment response, disease progression, and survival (Onyshchenko et al., 2022). Notably, early decreases in ctDNA levels has been associated with favorable outcomes, often preceding radiological findings. Our findings highlight the potentialities of FLARE in detecting longitudinal dynamics and identifying biomarkers to guide personalized schemes in HNSCC (Huang et al., 2023).

## 2 Methods

### 2.1 Plasma cfDNA samples, library construction, and sequencing

The NIVACTOR (EudraCT 2017-000562-30) trial was a single-arm, open-label, multicenter phase IIIb study evaluating nivolumab in 127 patients with recurrent or metastatic, platinum-refractory HNSCC. Patients received nivolumab 240 mg every 2 weeks until disease progression or unacceptable toxicity. The study assessed safety, objective response rate (ORR), progression-free survival (PFS), and overall survival (OS). Conducted in accordance with the Declaration of Helsinki and Good Clinical Practice guidelines, the study received approval from the local ethics committees. Translational analyses revealed that the Cl6 immune gene-expression signature identified patients having benefit from the treatment, highlighting a potential biomarker of nivolumab response (Serafini et al., 2024). Plasma was collected at baseline (C1D1) and at the beginning of fifth cycle of immunotherapy (C5D1) in our Institute (i.e., Fondazione IRCCS Istituto Nazionale dei Tumori). cfDNA has been extracted from either 500ul to 1000ul of plasma sample, based on material availability, following QIAamp MinElute cfDNA Mini protocol (QIAGEN). Extracted cfDNA has been quantified using Qubit Fluorimeter 2.0 DS High Sensitivity assay kit. CfDNA size and percentage compared to genomic DNA has been assessed using Tape Station Cell-free DNA ScreenTape. ONT libraries were made starting from 6 ng of cfDNA with native barcoding kit 24 v14 (SQK-NBD114.24) following the manufacturer’s protocol. Ten samples were multiplexed and sequenced on PromethION with flow cell FLO-PROM114M for 72h. The sequencing settings on the minKNOW software were the standard one, with basecalling and demultiplexing off.

### 2.2 Pre-processing

The raw Nanopore signals were subjected to basecalling using dorado basecaller (v 0.7.4) in HAC (high accuracy) mode with the--no-trim option to preserve complete reads without adapters or end trimming. Barcoded samples were subsequently demultiplexed using the dedicated dorado demux function, which separates reads according to their barcode sequences and generates per-sample output files suitable for downstream processing. The reads were then aligned to the human reference genome (hg38.p13) using minimap2 (Li, 2018) with the map-ont parameter, including the--MD tag and the -L option to include long CIGAR strings. The alignments were subsequently filtered with samtools (v 1.19.2) to preserve only primary, mapped reads with a minimum mapping quality of 20, while excluding supplementary and secondary alignments. Reads longer than 700 bp were retained and the final filtered alignments were used for downstream analyses (Katsman et al., 2022).

### 2.3 Copy number analysis and tumor fraction estimation

Tumor fraction and copy number variations (CNVs) were estimated using the HMMcopy (v 1.50.0) and ichorCNA Bioconductor/R packages. The ichorCNA software (v 0.3.2) was used to perform the copy number analysis and estimate the ctDNA tumor fraction for both short‐ and long‐read data mapped to the human genome. Exceptions to the software’s default settings were as follows: (a) a custom panel of normals generated from shallow whole-genome sequencing was used; (b) restart values for the non-tumor fraction parameter were increased to 0.95, 0.99, 0.995, and 0.999; (c) the ichorCNA ploidy parameter restart value was set to 2; (d) subclonal copy number states were not included; and (e) the maximum allowed copy number was reduced to 3. The tumor fraction corresponding to the highest log-likelihood was reported (Adalsteinsson et al., 2017; van der Pol et al., 2023).

### 2.4 Fragmentomic analysis

For fragmentomic analysis, fragment length distributions and associated quality control (QC) metrics were generated using NanoPlot (v 1.46.1). Sequencing reads were then classified into three fragment-size classes: (a) short (<150 bp), (b) intermediate (150–300 bp), (c) long (>300 bp) fragments. This size stratification was designed to test whether the shorter cfDNA fraction, often relatively enriched for tumor-derived molecules, contributes disproportionately to circulating tumor DNA (ctDNA) signals, consistent with prior observations that size selection can increase apparent tumor fraction in cfDNA analyses. For each fragment-size classes, tumor fraction and CNVs for these fragments were recalculated using HMMcopy and ichorCNA, applying the same parameter settings as described above to ensure direct comparability across size bins. This re-estimation enabled assessment of (i) how inferred ctDNA fraction changes when restricting analyses to specific fragment-length subsets and (ii) whether CNV profiles are stable or become more detectable as fragment size decreases, thereby supporting the investigation of size-dependent fragmentation patterns that have been reported as informative features of cfDNA in cancer settings. To compare tumor fraction estimates obtained from short fragments versus all fragments, a paired Wilcoxon signed-rank test was performed (Adalsteinsson et al., 2017; van der Pol et al., 2023).

### 2.5 Tumor fraction estimation with CancerDetector

Sorted and indexed BAM files generated by the ONT Dorado basecaller were used as input. These BAMs contain per-read methylation information encoded in the MM (modified base positions) and ML (modification likelihood/probability) tags. Standard BAM preprocessing was applied to ensure correct genomic ordering and enable random access. Using the *pysam* library (v0.22.0), alignments were parsed to extract modified cytosine positions from MM tags and their corresponding methylation probabilities from ML tags. Reads mapping to non-autosomal chromosomes or lacking valid MM/ML tags (or containing malformed/unsupported tag structures) were removed to prevent spurious methylation summaries.

Methylation calls were mapped to genomic coordinates and intersected with a reference panel of cancer-specific markers (*markers_panel.bed*). Mean methylation levels were computed for each marker region, generating a marker-by-sample methylation summary. These values were decomposed into tumor-associated and normal components and exported in tab-delimited format compatible with cfTools (Hu et al., 2025). Tumor fraction estimation was performed using the CancerDetector, which models marker methylation as a mixture of beta distributions for tumor and normal cfDNA. Marker-specific beta distribution parameters (α, β) were derived from training data, and posterior likelihoods were maximized under a regularization parameter (λ = 0.5) to reduce sensitivity to non-informative markers.

The final output reports estimated tumor and normal cfDNA proportions for each sample, along with intermediate marker-level summaries for diagnostic inspection and quality control. This pipeline provides an automated, reproducible workflow from Dorado-generated alignments to quantitative tumor fraction estimation.

### 2.6 Motif analysis

End-motif analysis was performed to characterize sequence patterns at the 5′ends of cfDNA fragments. For each sample, 4-mer motifs were extracted from the 5′end of mapped reads using a custom Python pipeline (*motif_analysis.py*). Reads with soft-clipped or hard-clipped bases at the 5′end were removed to reduce technical artifacts from alignment ambiguity or read trimming. Strand-specific handling was implemented to maintain a consistent 5′→3′orientation: for forward-strand reads, the 4-mer motif was taken directly from the 5′end, while for reverse-strand reads, the 4-mer was extracted from the reference at the 3′genomic end and reverse-complemented. Reads containing ambiguous bases (N) were excluded to preserve motif interpretability.

Motif counts were normalized by the total number of retained reads per sample, yielding relative frequencies for all 256 possible 4-mer motifs. Normalized frequencies from all samples were combined into a single matrix with rows representing samples and columns representing motifs. Motifs not detected in a sample were assigned a frequency of zero, producing a complete, non-negative matrix suitable for downstream analysis.

To identify latent fragmentation profiles, the motif frequency matrix was decomposed using non-negative matrix factorization (NMF) via the *NMF* R package (v0.2.8) (Gaujoux and Seoighe, 2010), applying a multiplicative update algorithm with random initialization (“vp”) and evaluating stability over 100 runs. The optimal factorization rank was determined empirically, with k = 3 selected. The factorization yielded two matrices: W, representing the contribution of each profile to individual samples, and H, representing motif composition of each profile.

Profile-defining motifs were identified by examining motif weights in H, and sample-wise contributions from W were compared across biological conditions. Paired comparisons (e.g., pre-treatment vs. post-treatment) were assessed using the Wilcoxon signed-rank test, in line with the study design. This approach follows previously validated cfDNA fragmentation frameworks (Katsman et al., 2022), ensuring methodological robustness and comparability.

### 2.7 Statistical analyses

All statistical analyses and visualizations were performed using the dplyr (v 1.1.4) and tidyr (v 1.3.1) packages for data processing, ggplot2 (v 4.0.0) and ggpubr (v 0.6.1) for data visualization, and stringr (v 1.5.2) for sample identifier parsing.

## 3 Results

### 3.1 Tumor fraction (longitudinal analysis of tumor fraction and cfDNA fragment size distribution)

**FIGURE 1:**
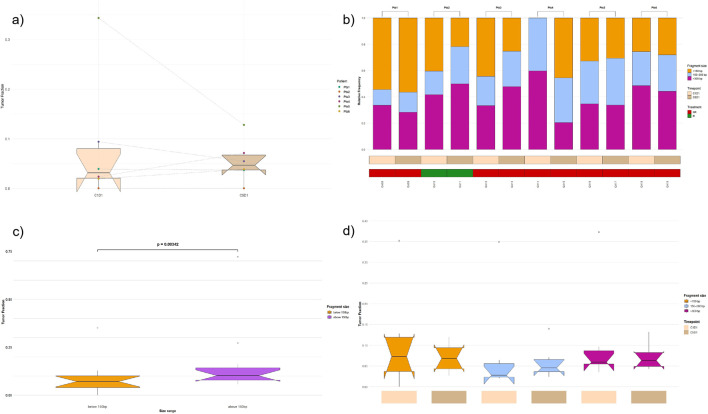
**(a)** Tumor fraction estimates across time points. Boxplots showing the estimated circulating tumor DNA (ctDNA) fraction in plasma samples collected at cycle 1 day 1 (C1D1) and cycle 5 days 1 (C5D1) from six patients. Tumor fraction was inferred from genome-wide copy number variation (CNV) profiles using ichorCNA across all cfDNA fragments. Individual colored points represent patient-specific estimates, with dashed lines connecting paired samples from the same patient to illustrate longitudinal changes. Boxes indicate the interquartile range, the center line denotes the median, and whiskers represent the data range. **(b)** Tumor fraction by cfDNA fragment size. The stacked bar plot shows the relative contribution of cfDNA fragments stratified by size (<150 bp, 150–300 bp, and >300 bp) across the twelve plasma samples. Objective response rate (ORR) was evaluated according to RECIST criteria (V.1.1). **(c)** Distribution of fragment proportions below and above 150 bp. Box-and-whisker plots show the median, interquartile range, and full data spread, with individual outliers indicated. Fragments >150 bp exhibit higher median values and greater variability compared with fragments <150 bp. **(d)** Distribution of fragment proportions by size class and experimental condition. Box-and-whisker plots summarize patient-level values for fragments <150 bp, 150–300 bp, and >300 bp at C1D1 and C5D1 (n = 6 patients).

Twelve plasma samples from six patients were analyzed, each with paired samples collected at two different time points: C1D1 and C5D1. The tumor fraction was estimated using ichorCNA. Overall tumor fraction across all cfDNA fragments have a median of 0.0685%, ranging from 0% to 0.346% with no significant difference between C1D1 and C5D1 (Figure 1a).

After stratifying fragments by size (<150 bp, 150–300 bp, and >300 bp), the relative contribution of each size class varies markedly among samples. Short fragments (<150 bp) dominate accounting relative frequencies >40% at both C1D1 and C5D1 of Pts 1 while in C1D1 of Pts 2 and Pts 3 and C1D5 of Pts 4. In contrast, Pts 5 and Pts 6 display a reduced proportion of short fragments and a higher contribution from medium and high length. Notably, C1D1 in Pts 4 lacks fragments below 150 bp and is characterized by an increased fraction of fragments larger than 150 bp. Across most samples, fragments above 300 bp contribute a substantial and relatively stable proportion, typically ranging from 30% to 50%. Overall, the data indicate pronounced patient variability in fragment size distribution, with no single size class consistently dominating across all samples (Figure 1b). Comparing the relative abundance of short (<150 bp) and longer (>150 bp) fragments across all samples, FLARE identified a significant association (p = 0.00342) as follow: i) fragments below 150 bp show a mean tumor fraction of 0.0899 (range 0–0.3517), consistent with previous observations in liquid biopsy studies in tumor malignancies (Mouliere et al., 2018); ii) fragments above 150 bp display a higher mean fraction of 0.1654 (range 0.05819–0.7218). Outliers are observed in the >150 bp group, suggesting increased sample-to-sample variability for longer fragments (Figure 1c).

Importantly, the higher mean tumor fraction observed in the >150 bp group was driven by a limited number of samples and was associated with substantially greater dispersion. In contrast, tended to show higher tumor fraction values across samples, in agreement with previous observations reporting enrichment of tumor-derived cfDNA among shorter fragments. Overall, these findings are consistent with a biological contribution of fragment size–dependent effects, although the limited sample size should be considered when interpreting these results (Tsui et al., 2025; van der Pol et al., 2023).

The distribution of fragment proportions across three size classes (<150 bp, 150–300 bp, and >300 bp) at two experimental conditions (C1D1 and C5D1) were assessed through FLARE and aggregated across the six patients. For fragments <150 bp, values at C1D1 display greater variability compared with C5D1. In the 150–300 bp range, both conditions exhibit relatively low median values, although C1D1 shows a wider spread and a prominent high-value outlier, indicating substantial inter-patient heterogeneity. For fragments >300 bp, distributions at C1D1 and C5D1 are broadly comparable, with moderate variability and overlapping interquartile ranges, though slightly higher dispersion is observed at C1D1. Overall, the box plots highlight pronounced inter-patient variability across all fragment size classes, with the largest spread consistently observed at C1D1, particularly for shorter and intermediate fragment lengths reflecting treatment effects and tumor dynamics in plasma (Figure 1d).

**FIGURE 2:**
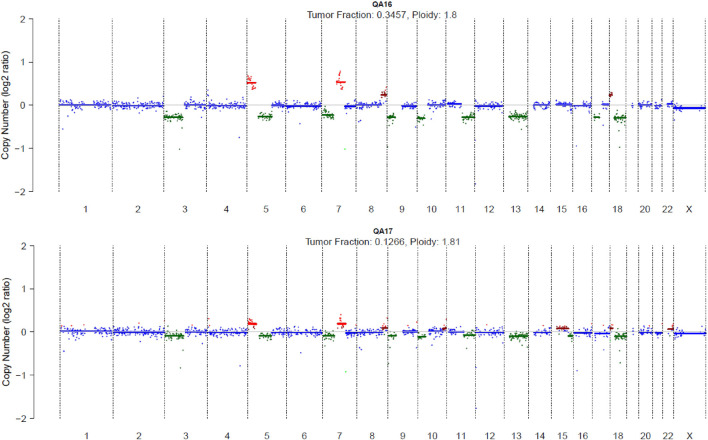
Genome-wide copy number profiles at C1D1 and C5D1. Scatter plots show copy number log2 ratios across chromosomes 1–22 and X. Colored points indicate copy number gains and losses relative to baseline, with segmented copy number states overlaid. Estimated tumor fraction and ploidy are reported for each time point, highlighting a marked reduction in tumor-derived DNA from C1D1 to C5D1.

Genome-wide CNV profiles revealed variable chromosomal alterations, including recurrent copy gains on 3q and 8q and losses on 9p21, consistent with known features of squamous carcinoma. FLARE identified an index case (Pts 5); at C1D1, the profile displays multiple focal copy number alterations, including both gains and losses across several chromosomes, consistent with a relatively high tumor-derived DNA contribution. This is reflected by the estimated tumor fraction of 0.3457 and an inferred ploidy of 1.8. In contrast, the C5D1 profile appears markedly flatter, with fewer and lower-amplitude deviations from baseline, indicating a substantial reduction in detectable copy number alterations following treatment. Correspondingly, the estimated tumor fraction at C5D1 decreases to 0.1266, while ploidy remains stable (1.81). Together, these data indicate a pronounced decrease in tumor-derived genomic signal over time, with overall genomic architecture remaining largely unchanged (Figure 2).

**FIGURE 3:**
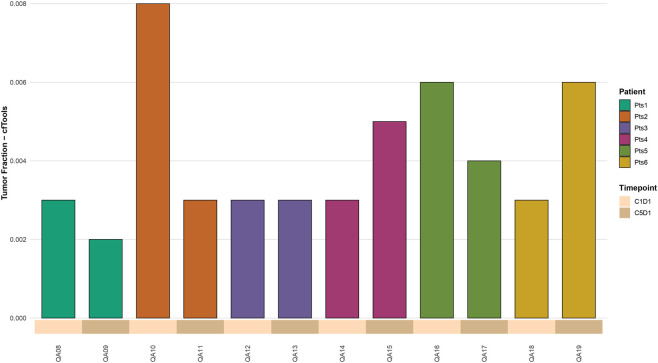
Barplots showing the estimated circulating tumor DNA (ctDNA) fraction in plasma samples collected at cycle 1 day 1 (C1D1) and cycle 5 days 1 (C5D1) from six patients using cfTools.

To further investigate, we applied the cfTools package to estimate tumor fraction based on CpG methylation sites. Estimated tumor fractions ranged from 0.0020 to 0.0080. The results showed a reduction in tumor fraction in three patients, with two displaying concordant trends with ichorCNA. The patient with an undetectable tumor fraction by ichorCNA exhibited a value of 0.0080 at C1D1, which decreased to 0.0030 at C5D1. One patient showed an increase in tumor fraction, also in agreement with ichorCNA, while the remaining two pairs showed no significant differences between pre- and post-treatment samples (Figure 3).

cfTools enables detection of tumor-derived cfDNA even at very low levels by leveraging CpG methylation signals, thereby increasing sensitivity compared to CNV-based methods such as ichorCNA. In samples showing more pronounced longitudinal changes, agreement between cfTools and ichorCNA was primarily qualitative, reflecting consistent directional trends rather than absolute concordance.

To further assess the agreement between methods, a quantitative comparison was performed between tumor fraction estimates obtained with ichorCNA and cfTools. Spearman correlation analysis did not reveal a significant monotonic correlation between the two approaches (ρ = −0.28, 95% CI −0.80 to 0.37, p = 0.38), indicating limited concordance in absolute estimates across samples. This result likely reflects both the small sample size and the different biological signals underlying CNV- and methylation-based inference.

The observed longitudinal changes in tumor fraction are presented as descriptive examples of potential changes in cfDNA-derived signals over time. These findings support the technical feasibility of tracking tumor fraction dynamics using cfDNA, while no clinical interpretation or predictive implications are inferred from this dataset.

### 3.2 5′-end nucleotide composition and fragmentation motif profiles of cfDNA

**FIGURE 4:**
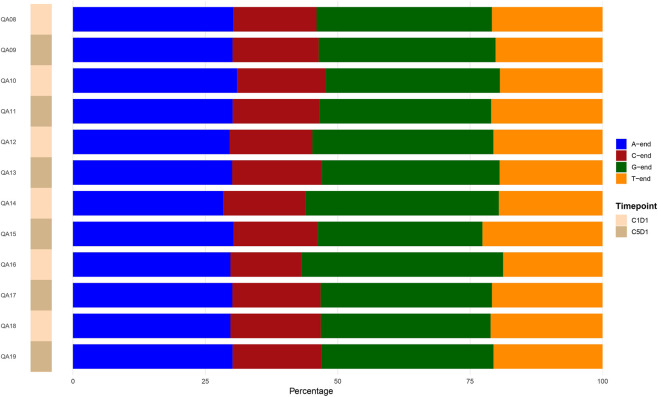
Barplots illustrate the percentage fraction in plasma fragment-end nucleotide composition across all samples.

The overall nucleotide composition at the 5′ends of cfDNA fragments was highly consistent across samples. In both sample groups (C1D1 and C5D1), fragments preferentially terminated with A and G nucleotides, whereas C-end fragments were consistently underrepresented (Figure 4). Across all samples, A-end frequencies ranged from 28.4% to 31.0%, G-end frequencies from 31.1% to 38.1%, C-end frequencies from 13.4% to 17.0%, and T-end frequencies from 18.8% to 22.7% (see Supplementary Table S1). Accordingly, no significant differences in 5′-end nucleotide composition were observed between C1D1 and C5D1 samples after Wilcoxon rank-sum analysis.

To further investigate sequence-specific fragmentation patterns underlying this stable nucleotide composition, we performed motif analysis of the 5′ends of cfDNA fragments by examining 4-mer motifs corresponding to the first four nucleotides of aligned reads. This approach is biologically motivated by evidence showing that cfDNA fragmentation is governed by specific nucleases, including DNASE1, DNASE1L3, and caspase-activated DNase (CAD/DFFB), each of which generates characteristic cleavage signatures (Serpas et al., 2019; Han et al., 2020). Using non-negative matrix factorization (NMF), three distinct profiles of terminal motifs were identified. Across all profiles, motifs enriched for A-end and G-end sequences predominated. Notably, A-end–enriched motifs were consistently observed, a pattern previously associated with CAD/DFFB-mediated cleavage during apoptosis (Han et al., 2020; Zhou et al., 2023), suggesting that cfDNA fragmentation in these samples is largely driven by apoptotic processes or high cellular turnover, which are known to dominate tumor-derived cfDNA release.

In contrast, motifs associated with DNASE1L3 activity—such as C-end–enriched patterns, including the canonical “CCCA” motif reported in healthy plasma—were largely absent (Serpas et al., 2019; Zhou et al., 2023). The lack of DNASE1L3-associated hallmarks supports the presence of an altered cfDNA fragmentation landscape, consistent with a predominant contribution of tumor-derived DNA to the circulating cfDNA pool. Comparison of matched pre- and post-treatment samples revealed no significant changes in the qualitative composition of the identified terminal motif profiles, in agreement with previous observations indicating that cfDNA fragmentation patterns primarily reflect underlying biological fragmentation mechanisms rather than short-term or treatment-induced effects (Zhou et al., 2023; Snyder et al., 2016)

## 4 Discussion

In this study, we introduce FLARE as an integrated pipeline for cfDNA fragmentomic analysis optimized for long-read Nanopore sequencing. By combining CNV profiling, tumor fraction estimation based on genomic and epigenomic signals, and end-motif analysis, we demonstrate the feasibility of extracting complementary fragmentomic features from Nanopore cfDNA data within a unified analytical framework.

A key aspect of FLARE is the adaptation of cfDNA analysis strategies originally developed for short-read sequencing to the specific characteristics of Oxford Nanopore data. Long-read sequencing preserves native cfDNA fragment lengths and enables direct detection of DNA methylation without bisulfite conversion or PCR amplification, allowing simultaneous investigation of fragmentation, copy number alterations, and methylation patterns from the same dataset.

Tumor fraction estimates derived from CNV analysis using ichorCNA revealed heterogeneous levels of cfDNA across patients and time points, consistent with variable cfDNA release in HNSCC. Due to the very limited number of patients, these observations should be interpreted as illustrative examples rather than evidence of a statistical association between cfDNA dynamics and treatment response.

Fragment size stratification confirmed enrichment of tumor-derived DNA in shorter cfDNA fragments, supporting fragment length as an informative feature for cfDNA characterization. Furthermore, methylation-based tumor fraction estimation using cfTools enabled detection of tumor-derived cfDNA at very low levels, including samples with limited CNV signal, highlighting the complementarity of genomic and epigenomic approaches.

The integration of CNV- and methylation-based tumor fraction estimation represents a key methodological aspect of FLARE framework. While ichorCNA and cfTools rely on fundamentally different biological signals (i.e., copy number alterations and CpG methylation, respectively) their combined use allows complementary characterization of tumor-derived cfDNA. In this study, no significant correlation was observed between tumor fraction estimates obtained with the two methods and these results should be interpreted in light of the limited cohort size and the exploratory, proof-of-concept nature of the work. Notably, qualitative agreement in longitudinal trends was observed in samples with a more pronounced tumor burden, while whereas discrepancies were more frequent at very low tumor fractions, where estimation becomes inherently more challenging.

To further explore cfDNA fragmentation biology, end-motif analysis characterized 5′-end sequence patterns, identifying motifs dominated by A- and G-end sequences, consistent with CAD/DFFB-associated apoptotic cleavage. The absence of DNASE1L3-associated motifs, typically enriched in healthy plasma cfDNA, supports an altered fragmentation landscape in tumor-derived DNA. The qualitative stability of motif profiles across paired samples suggests that end-motif signatures primarily reflect underlying fragmentation mechanisms rather than temporal variation.

Overall, these results demonstrate that long-read Nanopore sequencing can be effectively applied to multi-layer cfDNA fragmentomic analysis. The modular and reproducible design of FLARE provides a foundation for future methodological extensions and supports systematic investigation of cfDNA fragmentation features in cancer.

## 5 Conclusion

We developed FLARE, a scalable cfDNA analysis pipeline that integrates CNV profiles, tumor fraction estimates from both ichorCNA and methylation-based cfTools, and motif analysis with nuclease activity. This modular framework enables detailed and reproducible characterization of cfDNA fragmentomic features, providing a flexible platform for methodological development and technical benchmarking.

Fragmentomics analysis remains an emerging field, and we are actively expanding FLARE to address new challenges and enhance sensitivity, leveraging the unique advantages of Nanopore sequencing for epigenetic profiling.

## Data Availability

The datasets presented in this study can be found in online repositories. The names of the repository/repositories and accession number(s) can be found below: https://www.ncbi.nlm.nih.gov/geo/, GSE317007.
